# Microsurgical revascularization of a symptomatic proximal vertebral artery: pilot experiences from a single center

**DOI:** 10.3389/fneur.2023.1202565

**Published:** 2023-07-07

**Authors:** Tongfu Zhang, Donglin Zhou, Yangyang Xu, Maogui Li, Jianfeng Zhuang, Hai Wang, Weiying Zhong, Chao Chen, Hong Kuang, Donghai Wang, Yunyan Wang

**Affiliations:** ^1^Department of Neurosurgery, Qilu Hospital of Shandong University, Cheeloo College of Medicine and Institute of Brain and Brain-Inspired Science, Shandong University, Jinan, Shandong, China; ^2^Department of Neurosurgery, Yangxin County People’s Hospital, Binzhou, China; ^3^Department of Neurosurgery, Yantai Penglai People’s Hospital, Yantai, China; ^4^Department of Neurosurgery, The First Affiliated Hospital of Shandong First Medical University, Jinan, China; ^5^Department of Neurosurgery, The Second Affiliated Hospital of Guangxi Medical University, Nanning, Guangxi, China

**Keywords:** vertebral artery stenosis, vertebral artery occlusion, microsurgical revascularization, vertebral endarterectomy, in-stent restenosis

## Abstract

**Background:**

Vertebral artery stenosis and occlusion (VASO) is a high-risk factor for posterior circulation stroke. Post-stent restenosis and drug tolerance have facilitated the exploration of microsurgical vascular reconstruction. This study aims to evaluate the safety and efficacy of microsurgical reconstruction of the proximal VA.

**Methods:**

Twenty-nine patients (25 men, aged 63.2 years) who had symptoms of posterior circulation ischemia underwent microsurgical revascularization for proximal VASO were retrospectively included in this study. Procedural complications and clinical and angiographic outcomes were reviewed.

**Results:**

Twelve, three, and five patients underwent VA endarterectomy, artery transposition, or both, respectively; seven patients underwent vertebral endarterectomy plus stent implantation; and two patients failed surgery because of the difficult exposure of the VA and the occurrence of vascular dissection. The perioperative period-related complications included seven cases of Horner’s syndrome, five cases of hoarseness, and one case of chylothorax. No cases of perioperative stroke or death were reported. The mean follow-up period was 28.4 (8–62 months). Most patients improved clinically; however, the vertebrobasilar ischemia symptoms did not decrease significantly in two patients during the follow-up. Moreover, follow-up imaging was performed in all the patients, and no signs of anastomotic stenosis were reported.

**Conclusion:**

Microsurgical reconstruction is an alternative option that can effectively treat refractory proximal VASO disease and in-stent stenosis, with a high rate of postoperative vascular recirculation. Prospective cohort studies with larger sample sizes must be conducted to validate the above conclusions.

## Introduction

Cerebral stroke is a common disabling fatal neurological disease. Studies have shown that approximately 25–40% of strokes occur in the posterior circulation (1–4). The mortality rate of posterior circulation strokes (33%) is higher than that of anterior circulation strokes (20–30%) (5). Previous studies have indicated that approximately 20% of posterior circulation ischemia cases are caused by VASO (6–9), which occurs in 5% of the normal population (10). However, with the development of imaging techniques, proximal VASO has been highly diagnosed.

Moreover, proximal VASO is treated with intensive drug therapy, including antiplatelet and lipid-lowering therapies. Surgical treatment may be considered for patients with poor response to drug therapy or recurrent cerebral infarction after therapy. Endovascular treatments such as balloon dilation or stenting have become the leading surgical modalities for proximal VASO because of their simplicity, low perioperative complications, and high success rates. However, high restenosis rate (up to 30%) is an essential drawback of endovascular treatment (11, 12). Although drug balloons or drug-eluting stents may reduce restenosis, they are still associated with higher restenosis rates of 11–30% as previously reported (13, 14).

In addition, not all patients with proximal VASO, including those with chronic occlusion or in-stent stenosis of the proximal VA, are eligible for endovascular treatment. Moreover, stent implantation may lead to irreversible complications in patients with severely curved proximal VA. These drawbacks have encouraged the exploration of microsurgical revascularization of proximal VA. It comprises two main methods: vertebral endarterectomy (VEA) and bypass transposition such as VA-common carotid artery (CCA), VA-subclavian artery (SA), and VA-thyroidal trunk transpositions (5, 12). Revascularization of the proximal VA has been widely used as a traditional surgical method in the treatment of VASO before the development of interventional techniques (14). Although these procedures are relatively traumatic, risky, and limited by medical conditions, previous studies have revealed that they can effectively reduce symptoms associated with ischemia of the brain. Advances in endovascular techniques have hindered the development and application of these techniques. However, endovascular therapy cannot completely replace revascularization techniques, as both have advantages and limitations. Recently, there have been only a few reports on these surgeries. Moreover, the experience and skill levels of surgeons are different. In this study, we describe the microsurgical reconstruction of a symptomatic proximal VASO at our center. Safety and efficacy were assessed through follow-up of postoperative complications and improvement of imaging modalities.

## Materials and methods

### Summary of case

The study was approved by the Medical Ethics Committee of Qilu Hospital of Shandong University. We retrospectively analyzed the clinical data from November 2016 to May 2021 of 29 patients with severe VASO and restenosis of the VAs after stent implantation at Qilu Hospital of Shandong University. The inclusion criteria were as follows: (1) severe stenosis (≥70% diameter reduction) of the unilateral VA, moderate stenosis (50–70% diameter reduction) of the dominant VA when the contralateral was hypoplastic or occluded, or the termination of the posterior inferior cerebellar artery; stenosis was confirmed by computed tomography angiography (CTA) or magnetic resonance angiography (MRA); (2) symptoms refractory to medical treatment, including dual antiplatelet therapy, statin therapy, blood pressure, and glucose control; (3) restenosis after stenting of the proximal VA; and (4) patients who refused proximal VA endovascular treatment.

The exclusion criteria were as follows: (1) severe cardiac or pulmonary failure; (2) VASO caused by conditions rather than atherosclerosis, such as extrinsic compression, dissection, radiation arteritis, or fibromuscular dysplasia; and (3) severe bleeding tendency. We collected patients’ demographic characteristics data (age and sex), medical comorbidities (diabetes mellitus, hypertension, and hyperlipidemia), radiographic features, microsurgical revascularization methods, procedural complications, and clinical and imaging follow-up. The patients’ morning fasting venous blood samples were obtained within 24 h of admission. Triglyceride, cholesterol, low-density lipoprotein, and high-density lipoprotein levels were measured. Magnetic resonance imaging (MRI) was performed before treatment to confirm infarction or the insufficiency of the vertebrobasilar territory. Stenosis was confirmed using digital subtraction angiography or CTA. Stenosis was considered severe when the angiographic diameter was narrowed by 70% or more. In this study, complications lasting for 30 days or less were considered temporary. Clinical follow-up was performed via clinical examination or telephone interviews, with a median follow-up time of 23 months (4–66 months) for the surviving patients. The patients’ pre- and postoperative neurological functions were evaluated using the modified Rankin Scale (mRS) scores. Radiological follow-up was performed using CTA, MRA, or digital subtraction angiography (DSA) 6–30 months after treatment. Restenosis was defined as stenosis with a diameter reduction of more than 50% or an occlusion.

### Surgical technique

After general anesthesia, patients were placed in a supine position with their head tilted to the opposite side of the diseased VA to fully expose the anterior cervical area. An L-shaped incision was made in the neck from the anterior edge of the sternocleidomastoid muscle to the sternocleidomastoid joint and extended along the collarbone to the lateral portion of the neck. The platysma muscle was cut to separate the hyoid and peritracheal muscles, consistent with the standard carotid endarterectomy (CEA). The jugular vein was pulled outward into the carotid sheath. The CCA and internal jugular vein were separated, and the posterior cervical fascia was exposed.

**Figure 1:**
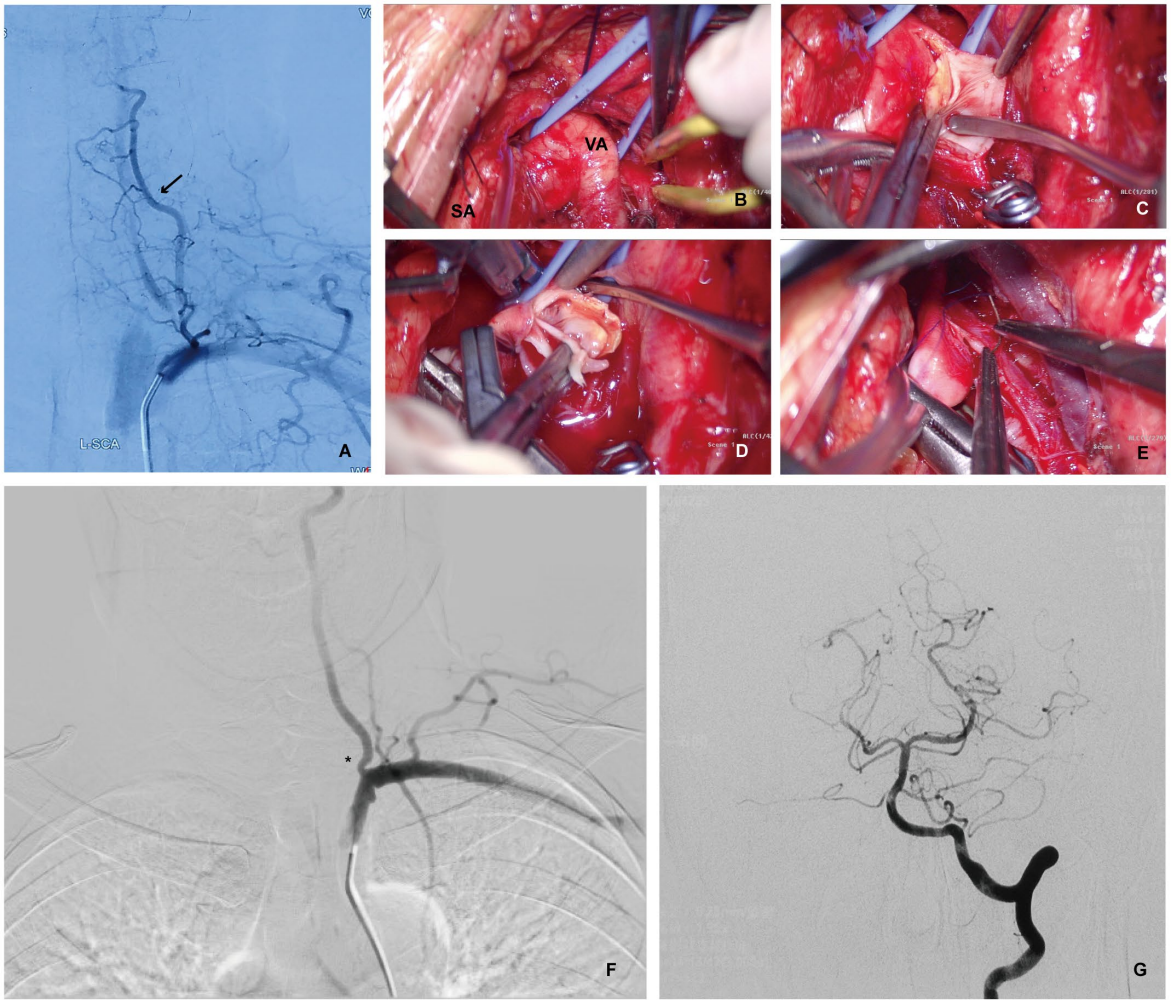
Patient 1 with recurrent transient ischemic attacks of the vertebrobasilar artery despite reasonable medical therapy. **(A)** Digital Subtraction Angiography (DSA) showing complete occlusion of the left VA with blood flow countercurrent into the left VA via the thyrocervical trunk (arrow). **(B)** Intraoperative photograph showing left SA and VA exposure via the supraclavicular approach for a left VEA. **(C)** Intraoperative photograph showing a longitudinal VA incision to expose atherosclerotic plaque after the SA and distal VA were temporarily blocked using aneurysm clips. **(D)** Intraoperative photograph showing a left VEA. **(E)** Intraoperative photograph showing the dissection of the left VA plaque, and suture incision. **(F,G)** Postoperative DSA showing significant improvement on vessel diameter and improved flow (asterisk). No posterior circulation vessel abnormalities were found.

**Figure 2:**
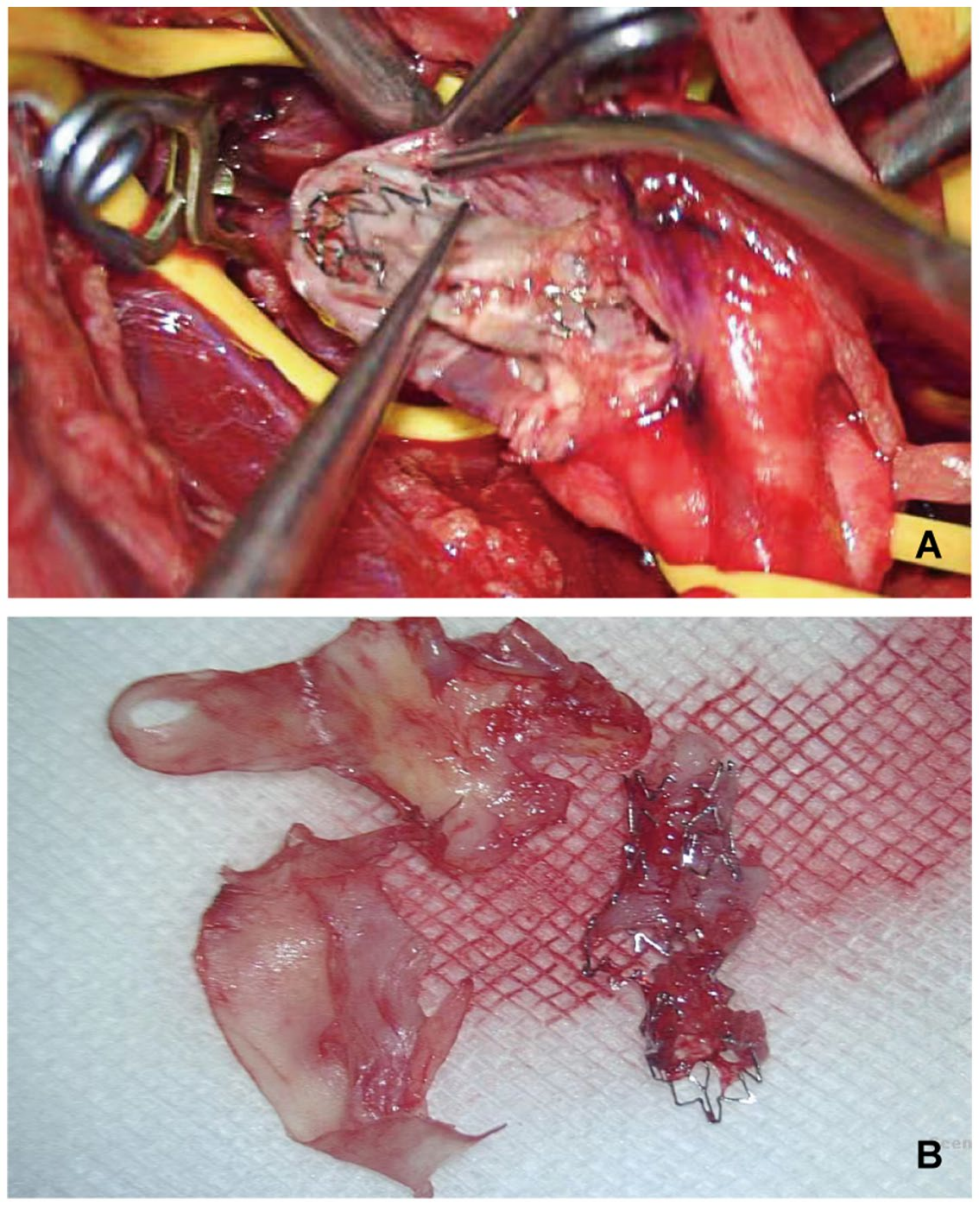
Patient 2 with restenosis of right VA stent, undergoing right VEA and stent removal. **(A)** Intraoperative photograph showed a longitudinal incision of the vertebral artery, showing interlacing of stent and arterial plaque. **(B)** The photograph showing post-operative stripping of arterial plaque and rupture stent.

The transverse process of the sixth cervical vertebra, which serves as a marker for the VA entering the transverse foramen, was identified by palpating the carotid tubercle, and the VA was identified by exploring the space between the anterior scalene and longus colli. The vertebral vein is located anteriorly; thus, care should be taken to avoid tearing its branches while pulling. The thyroid trunk and internal thoracic artery must be separated and protected (in preparation for reconstruction) when dissecting the VA distal to the SA. The cervical sympathetic trunk is deep in the VA. Surgeons must carefully identify and ligate lymphatic vessels entering the subclavian vein, particularly the thoracic duct, whose branches are clearly observed on the left side during surgery, but the lymphatic vessels on the right side were generally small. In addition, the recurrent laryngeal nerve is an essential anatomical structure that is commonly observed in successful operations. After the intravenous administration of 5,000 units of low-molecular-weight heparin (LMWH), the vertebral and subclavian arteries were clamped using vascular or aneurysm clips. The VA was then longitudinally incised to the SA, which was ligated with 7–0 nylon sutures after the VEA (Figures 1, 2).

**Figure 3:**
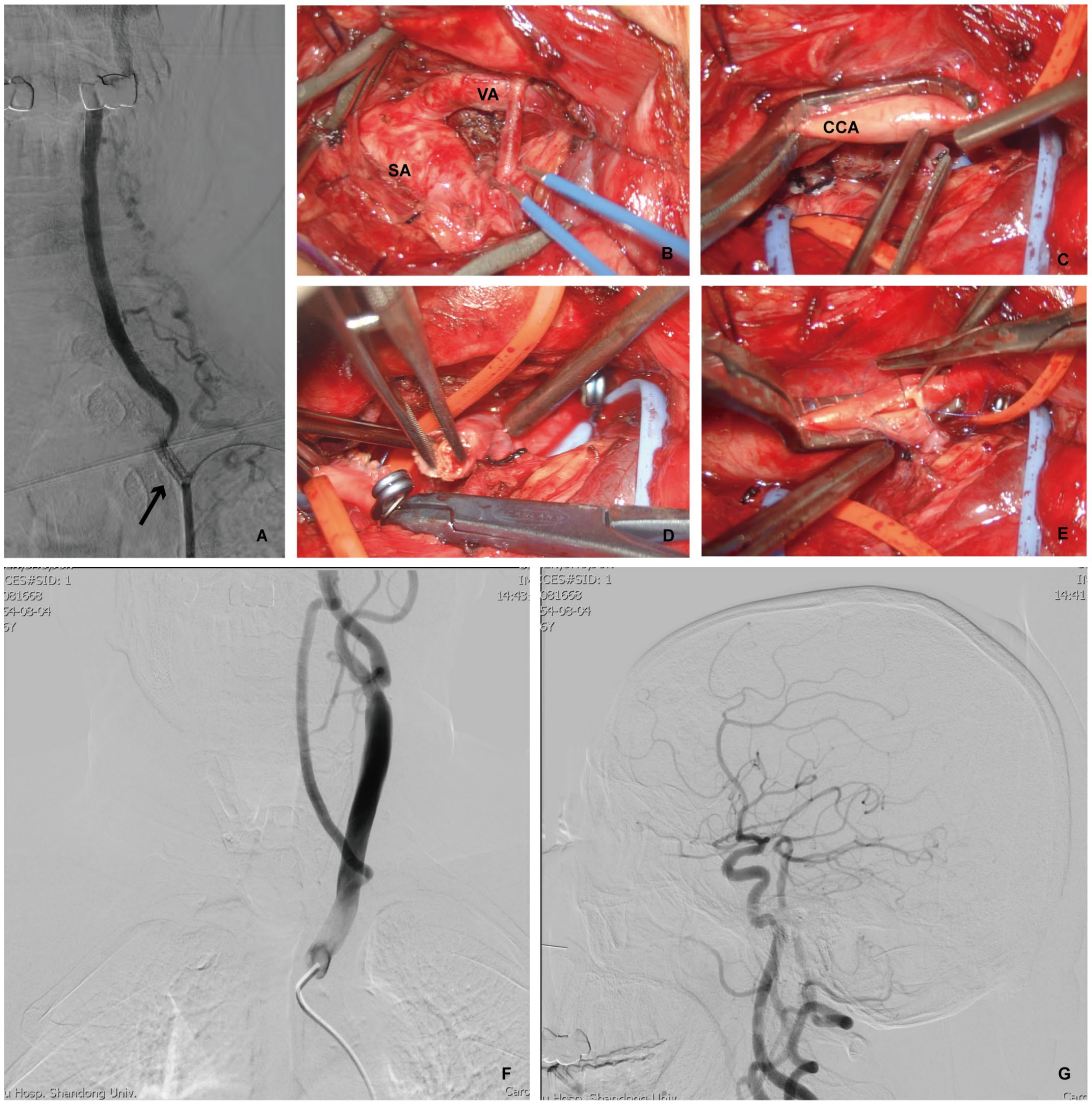
Patient 3 developed left VA opening stenosis again after left VA stent implantation, and underwent VA-to-CCA transposition. **(A)** DSA indicating restenosis after the left VA stent (arrow). **(B,C)** Intraoperative photograph showing adequately exposure of the left SA, VA and CCA via the supraclavicular approach. Intraoperative photograph demonstrating ligation and severing of the left VA **(D)**, then prepared **(E)** for left VA- to- CCA transposition. **(F,G)** Postoperative DSA, cervical view, illustrating the confirmed patency of the anstomosis with good intracranial flow.

It is difficult to expose the vertebral arteries sufficiently without removing a part of the collarbone in patients with low vertebral initiation. Vertebral transposition may be an appropriate treatment option. The VA was ligated as close as possible, and the clamped CCA was rotated to anastomose the posterior lateral wall. The CCA was incised using a vascular puncture device of the same diameter as that of the VA, and the VA was anastomosed using a 7–0 nylon suture. An anastomosis between the vertebral and subclavian arteries was considered in patients with tortuous proximal VAs. Indocyanine green (ICG) fluorescence imaging and angiography were used in all patients undergoing either VEA or revascularization to ensure a smooth arterial flow. Combined endovascular therapy or revascularization should be considered in patients in which VA plaques are difficult to expose or VEA fails. Blood pressure was maintained at high levels to increase perfusion during surgery (Figure 3).

We followed up the study participants for 1, 3, and 6 months after surgery, focusing on surgical complications and symptom reduction. If nerve damage occurred, mecobalamin was administered. A detailed neurological examination was performed, and vertebral artery flow was reviewed using cervical and intracranial vascular CTA. Repeat angiography was performed in patients with posterior ischemia or stroke.

## Results

A total of 29 consecutive patients with symptomatic proximal VASO were included. The sample consisted of 25 males and 4 females with a median age of 63.2 years (44–79 years). Among them, 19 patients were treated for the first time, and 10 had restenosis or occlusion after previous stenting. Comorbidities included hypertension in 26 patients (89.6%), diabetes mellitus in nine patients (31%), and hyperlipidemia in four patients (13.8%). Their basic clinical characteristics are shown in Table 1.

**Table 1:** Baseline clinical characteristics of patients (*N* = 29).

Parameter	Result
Age [years]	63.2 ± 8.5
Sex, male	25 (86.2%)
Vertebral artery stenosis (Left:Right)	18:11
Hypertension	26 (89.7%)
Diabetes mellitus	9 (31%)
Hyperlipidemia	4 (13.8%)
History of TIA	24 (82.7%)
Prior stroke	10 (34.2%)
Occipital lobe and pontine stroke	3 (10.3%)
Anterior circulation stroke	7 (24.1%)
Restenosis after stent implantation	10
Smoking status	11 (37.9%)
Current drinking	10 (34.5%)
Coronary artery disease	7 (24.1%)
Previous percutaneous coronary intervention	3 (10.3%)
Previous carotid endarterectomy	2 (6.9%)
Posterior traffic artery compensates for blood supply	2 (6.9%)
Contralateral vertebral artery occlusion	11 (37.9%)
Coexisting carotid artery stenosis	9 (31%)
Coexisting basilar artery stenosis	2 (6.9%)
mRS- preoperative	
0	0
1	19
2	10

TIA, Transient ischemic attack.

Clinical manifestations of vertebrobasilar system ischemia, such as vertigo, dizziness, posterior cranial nerve deficiency, ataxia, and hemispheric symptoms of the internal carotid artery, were also collected. Neurological deficits with posterior circulation infarction were confirmed in three patients, and vertebrobasilar insufficiency without infarction was confirmed in 26 patients. Among them, 15 (51.7%) had recurrent transient ischemic attack (TIA), and 7 (24.1%) had coexisting cerebral hemispheric symptoms. The preoperative (Modified Rankin Scale) mRS scores are presented in Table 2.

**Table 2:** Specific surgical method (*n* = 29).^a^

Method of operation	Result
VEA	12 (41.4%)
VEA and stent implantation	7 (24.1%)
VEA and transposition	5 (17.2%)
Transposition	3 (10.3%)
Failure of the operation	2 (13.8%)
All transposition surgery targets blood vessels	8 (27.6%)
VA-CCA transposition	4 (13.8%)
VA-SA transposition	3 (10.3%)
VA-thyrocervical trunk transposition	1 (3.4%)

^a^VEA, Vertebral endarterectomy; CCA, Common carotid artery; CEA, Carotid endarterectomy; SA, subclavian artery.

Preoperative angiography was performed for all patients. The target vessels were located on the left side in 18 patients and on the right side in 11 patients. The target vessel presented significantly severe stenosis (≥70%) in 21 patients (72.4%) and occluded stenosis in 8 patients (27.6%). The contralateral VA was occluded in 13 patients (44.8%), and severe or hypoplastic stenosis was present in four patients (13.8%). Coexisting internal carotid artery occlusion or stenosis was observed in nine patients. No cases of steal syndrome associated with subclavian occlusion were observed.

In this study, 27 patients (93.1%) underwent successful vertebral artery revascularization, 12 patients (41.4%) underwent simple VEA, three patients (10.3%) underwent simple transposition, and five patients (17.2%) underwent VEA combined with transposition. Seven patients had long VA stenosis segments. Distal stenting was undertaken after proximal VEA (Table 3). The difficulty of vertebral artery exposure and the vascular dissection after VEA were the main reasons for the failure of vascular reconstruction.

**Table 3:** Postoperative complications.

Complications	Transient no. (%)	Permanent no. (%)
Follow-up (months)	28.4 ± 18.1	
Hoarseness	7 (24.1%)	3
Dysphagia	3 (10.3%)	1
Honer’s syndrome	5 (17.2%)	0
Chylothorax	1 (3.1%)	0
In-stent thrombosis	1 (3.1%)	0
Minor cerebral hemorrhage	1 (3.1%)	0
Phrenic nerve palsy	0	0
TIA	0	0
mRS-Postoperative		
0	0	
1	10	
2	19	

### Complication and outcome

The mean follow-up time was 28.4 months (8–62 months). No new strokes or deaths were observed during follow-up. All patients completed clinical and radiological follow-ups. Imaging data included MRA and CTA. DSA was performed to determine the vascular status of the patients suspected of having surgically targeted vascular stenosis and newly developed vertebral arterial system symptoms.

The overall complication rate was 31% with a dominance of nerve injury. During the immediate postoperative period, Horner’s syndrome was recorded in five patients, but they recovered completely at the follow-up assessment. Postoperative hoarseness was recorded in seven patients, and it may be related to recurrent laryngeal nerve injury. All patients were treated with mecobalamin once nerve damage occurred. Based on a one-year follow-up phone interview, hoarseness persisted in three patients and dysphagia in one patient, although these symptoms had partially decreased. Four patients adapted to the neurological deficits that did not interfere with their daily lives. In addition, one patient developed a lymphatic fistula with chylothorax after surgery, but it was completely cured by continuous closed thoracic drainage. At the last follow-up evaluation, 19 patients (65.5%) had mRS scores of 1, and 10 patients (52.6%) had mRS scores of 2. This was consistent with the preoperative scores.

No cases of target vessel restenosis were observed on follow-up imaging. Of the patients who successfully underwent surgery, only two patients had no significant decrease in symptoms.

## Discussion

There is no cohort study data on improving stroke prevention through revascularization of the proximal VA versus existing therapies. According to our case study data, most patients received maximal medication or interventions but experienced adverse effects. Therefore, microsurgical vascular reconstruction was performed to improve vertebral artery flow.

Microsurgical revascularization for posterior cerebral circulatory ischemia has unique advantages. The extra-cranial arterial system is introduced directly into the ischemic brain tissue by re-establishing the collateral circulation pathway through surgery. This improves perfusion and prevents stroke recurrence (15–19). Previous studies also support our findings; Morasch reported that the 10-year patency rate was as high as 90% (20), while Rangel-Castilla et al. reported a success rate of approximately 93–98%, with only 5% of patients experiencing restenosis during follow-up (14). Therefore, microsurgical revascularization of the proximal VAs is an effective treatment and should be considered for patients with VASO. However, complications associated with this procedure are not negligible.

In our study, the overall procedural complication rate was 31%, while it ranged from 4.5 to 45.5% in a previous study (14). The most common complications were vocal cord paralysis and Horner’s syndrome. The cervical sympathetic nerve (CSN) is usually located anterior or ventral to the vertebral artery; thus, it may be difficult to completely expose the proximal VA without damage. Fortunately, most cases of Horner’s syndrome are temporary and recover gradually without long-term neurological defects. To minimize these complications, it is necessary to protect the recurrent laryngeal nerve during surgery to avoid tearing that leads to permanent hoarseness. The thoracic ducts should be carefully identified and ligated. We routinely provide drainage for 3–4 days after surgery to avoid the occurrence of chylothorax. Normal identification of the VA is also important to reduce operation-related complications. The VA is typically located deep in the vertebral vein. Unlike the thyroid neck trunk, the VA has no branches and continues to the C6 transverse process. This indicates that the intracranial VAs cannot be palpated during surgery. Traditional reconstructive surgery involves removing a part of the collarbone to completely expose the vertebral and subclavian arteries. However, this method is prone to poor healing and pleural damage, leading to pneumothorax (21). In conclusion, microsurgical revascularization should not be the first choice for symptomatic VASO treatment because of the deep narrow operating space and complex and difficult-to-expose anatomical structures. Although this technique has some limitations, it may be more suitable for patients with contraindications or difficulties with endovascular treatment.

In our study, endarterectomy without transposition was commonly performed (41.3%), unlike a previous study in which vertebral artery bypass transposition was more common (21). This may be because of the case selection and surgeon’s experience. We believe that endarterectomy alone offers more consistently reliable results with normal human vascular anatomy, and it may be more suitable for patients with easy-to-expose proximal VAs and proximal and distal subclavian arteries with a VA origin. Vertebral-subclavian artery bypass may be suitable for patients with a low-level VA origin or a tortuous proximal VA when the subclavian artery can be fully exposed and temporarily blocked. Vertebral-subclavian artery bypass also conforms to normal human vascular anatomy compared to VA-common carotid anastomosis, and there is no need to block the CCA temporarily during the operation, as this does not induce intraoperative plaque shedding and anterior circulation insufficiency postoperatively. VA-CCA anastomosis may be suitable for patients with hard-to-expose subclavian arteries, although this method has been widely used in previous studies. However, it may increase the risk of anterior circulation insufficiency, especially in patients who already have insufficient blood supply. There was one patient for which we performed vertebral-thyroid artery anastomosis, which has been rarely reported in the literature (14, 22). Compared to VA-CCA bypass, vertebral-thyroid artery anastomosis resembles normal anatomy - since the thyroid trunk also originates from the subclavian artery, and there are extensive collateral branches of the thyrocervical trunk originating from the external carotid- and less likely to cause anterior circulation insufficiency; thus, proved to be a valid alternative treatment. Combined microsurgical revascularization and endovascular treatment would increase the recanalization rate in patients with a high stenosis location and long VA stenotic segments. For lesions with completely occluded openings that cannot be opened directly through the lumen, a combined operation is safer and has a higher success rate than single-lumen operation.

Endovascular intervention can partially compensate for the limitations of reconstructive surgeries. First, we tried to temporarily occlude the SA with a balloon to reduce arterial damage and ensure a smooth VEA. However, post-operative angiography remains the gold standard in determining blood flow patency. Effective initial VEAs do not necessarily improve blood flow. Furthermore, the angiogram surprised us with uniform stenosis at the beginning of the VA in one of our successful VEAs. Presumably, atrophy of the vascular elastic fibers due to long-term plaque compression combined with a lack of plaque support after VEA led to vascular collapse and poor compliance. We increased the blood flow to the VA by confining the operative upper limb. This can be distinguished from vascular stenosis.

In-stent restenosis remains an intractable problem. Whether balloon dilation or stent re-implantation is used, the possibility of restenosis still exists after surgery. In covering an existing lesion with a new stent, there are difficulties with guide wire passage and stent release. In this case, surgical revascularization treatment seems to be the best option. Compared to plain atherosclerotic stenosis, VEAs in these patients are challenging. Firstly, it is essential that the distal and proximal ends of the stenosis lesions be fully exposed. Secondly, because the stent has been integrated into the plaque and wrapped by the outer membrane, it is easy to cause perforation of the vessel wall when the stent and plaque are stripped as a whole, making it difficult to completely preserve the outer membrane of the artery and increasing the difficulty of suturing. In addition, vessel walls are thinner and less elastic after stent removal, and there may be persistent blood leakage after suturing. Therefore, performing transposition surgery to reconstruct blood flow is also a good option for patients with difficult in exposing.

### Limitations

The findings in this report are subject to at least four limitations. First, this was a single-center study with a small sample size; a larger sample size would be more reliable for evaluating treatment effectiveness. Second, the inclusion of a heterogeneous group of patients with varied microsurgical revascularization may also have led to a selection bias. Third, the follow-up period was short, and not all included patients had the same imaging follow-up data. Fourth, there was no control group; a large-sample randomized controlled study would be more effective in evaluating the therapeutic effects of microsurgical revascularization on severe stenosis or occlusion of the proximal VA.

## Conclusion

Microsurgical revascularization of the proximal VA is an effective treatment for initial VASO but is associated with a high risk of local complications. Therefore, it may not be a suitable choice for VASO patients with contraindications or technological difficulties with endovascular treatment.

## Data availability statement

The original contributions presented in the study are included in the article/supplementary material, further inquiries can be directed to the corresponding author.

## Ethics statement

The studies involving human participants were reviewed and approved by the Ethics Committee of Qilu Hospital of Shandong University. Written informed consent for participation was not required for this study in accordance with the national legislation and the institutional requirements. Written informed consent was obtained from the individual(s) for the publication of any potentially identifiable images or data included in this article.

## Author contributions

YW and TZ: conceptualization. DZ and TZ: data curation and drafting manuscript. YX, ML, JZ, and HW: project administration, case collection, and operation. CC, HK and DZ: follow-up visit, supervision, and writing review and editing. YW and HK: funding acquisition, reviewing the manuscript and supervision. All authors have read and agreed to the published version of the manuscript, contributed to the article, and approved the submitted version.

## Funding

This research was supported by International Scientific Exchange Foundation of China (No. Z2018LSD001), the Basic Ability Enhancement Project of Young Teachers in Guangxi Zhuang Autonomous Region (No. 2021KY0089), and the Research Project of Guangxi Health and Family Planning Commission (No. Z20190651).

## Conflict of interest

The authors declare that the research was conducted in the absence of any commercial or financial relationships that could be construed as a potential conflict of interest.

## Publisher’s note

All claims expressed in this article are solely those of the authors and do not necessarily represent those of their affiliated organizations, or those of the publisher, the editors and the reviewers. Any product that may be evaluated in this article, or claim that may be made by its manufacturer, is not guaranteed or endorsed by the publisher.
